# Examining the Impact of COVID-19 Experiences on Reported Psychological Burden Increase in Older Persons: The Effects of Illness Severity and Social Proximity

**DOI:** 10.3389/fpsyg.2022.884729

**Published:** 2022-05-13

**Authors:** Theresa Heidinger, Lukas Richter

**Affiliations:** ^1^Department of Gerontology and Health Research, Karl Landsteiner University of Health Sciences, Krems an der Donau, Austria; ^2^Department of Social Sciences, St. Pölten University of Applied Sciences, St. Pölten, Austria

**Keywords:** social network, COVID-19, infection, severity, psychological burden

## Abstract

Previous findings have provided indications that experience of COVID-19 illness of self and other affect mental health unfavorably. However, prior studies do not satisfactorily differentiate according to severity of COVID-19 illness or social proximity, which are both hypothesized to be relevant factors for increased psychological burden. This study provides an in-depth examination of the impact of Covid-19 experience of self and other on mental health, considering illness severity as well as proximity to the infected person (self, close and distant network). It used data on an older population (50+ years) from 28 European countries (*n* > 40 000 persons) surveyed in summer of 2021 using the Survey of Health and Retirement in Europe (SHARE). Aside from bivariate analyses, a logistic regression model was computed to test the effects of illness severity by personal proximity over and above other stressors of life in the pandemic. Severity of illness was shown to be a contributor to psychological burden increase with the strongest effects among persons who reported own illness experiences or experiences in the close network. Regression analysis confirmed the impact of severe Covid-19 experience in self, close and distant relations. Moreover, even a less severe course impacted burden unfavorably when experienced in the own person and more distant relations. These results prove troubling. Psychological burden is impacted by infection, with experiences in self or close persons being strongest, while even ‘lighter' experiences in the distant network also have an unfavorable effect, emphasizing the need to gain control of the present pandemic.

## Introduction

With the global pandemic entering its third year more and more research is being conducted on the impacts of life during COVID-19 on physical and mental health. Large population studies report these impacts as overwhelmingly negative with social restrictions specifically being faulted for deteriorating well-being. This seems especially true for older persons, who are faced with higher likelihood of more severe course of disease as well as mortality (Gerwen et al., 2021) and continue to be marginalized by society in an attempt at protection via health policies asking them to remain isolated and/or physically distance themselves from others (Ehni and Wahl, 2020). These policies, which should prevent infection have had negative consequences on mental health such as increased subjective isolation (Peng and Roth, 2021) and loneliness (Krendl and Perry, 2021; Richter and Heidinger, 2021b)—well-known correlates of increased anxiety, depression and stress in older age (Courtin and Knapp, 2017). It is unsurprising, that these illnesses as well as sleeping disorders have reportedly increased among the older population during the pandemic (Bailey et al., 2021; De Pue et al., 2021; Grolli et al., 2021; Zaninotto et al., 2022).

In addition to unfavorable changes to older peoples' lifestyle during the pandemic such as isolation or cocooning it has been shown, that experiencing COVID-19 infection second hand, in the social network negatively effects well-being, with the severity of experience being predictive of psychological burden (feeling anxious, depressed or sleeping badly) over and above other difficulties of life during COVID-19 such as subjective loss of control, physical health or sociodemographic variables (Richter and Heidinger, 2021a). This result was in line with findings reported during the SARS pandemic (Hawryluck et al., 2004; Wu et al., 2009) as well as studies focussing on the early days of the COVID-19 pandemic which found increased anxiety, depression and PTSD in persons who had experienced Covid-19 illness—(Gallagher et al., 2020; González-Sanguino et al., 2020; Mazza et al., 2020; Park et al., 2020) or mortality (Breen et al., 2021) in the social circle. Although the previous paper by this research team included a multivariate model testing the effect of other-experience while controlling for further potential burdens of life during the pandemic, it did not differentiate between proximity levels to the infected party. It also omitted the important effect of own-experience of Covid-19 illness thereby limiting the interpretability of the effect of other-experience of COVID-19 illness^1^ (Richter and Heidinger, 2021a). The adverse effect of other-experience of illness has been reported previously (Gallagher et al., 2020; Mazza et al., 2020; Park et al., 2020) and can be interpreted using the concept of secondary traumatic stress defined as “*the natural, consequent behaviors and emotions resulting from knowledge about a traumatizing event experienced by a significant other. It is this stress resulting from helping or wanting to help a traumatized or suffering person*” (Figley, 1999, p. 10). This study will take on these points and provide an expanded view on the effect of severity of COVID-19 experience in self and others on mental health using more recent data (summer 2021).

Psychological burden or more precisely the reported increase in burden, as used in this study, will be measured using information on feelings of anxiety, depression, and troubled sleep which conjointly result in an unfavorable mental condition. Burden increase is assumed to be associated with the severity of the COVID-19 own experience but also when experienced second hand through other persons in the social circle. It is also likely be influenced by other factors of life during the pandemic. Poor health status has been found to be a risk factor for increased psychological burden with previous mental health issues (Blix et al., 2021) or concurrent illness (Shevlin et al., 2020; Wu et al., 2021) being connected to increased probability of depression, anxiety, sleep disturbance and distress in the pandemic. Additionally, feeling a subjective loss of control (i.e. an external locus of control) was found to be related to increased psychological distress during the pandemic (Sigurvinsdottir et al., 2020; Alat et al., 2021). Furthermore, sociodemographic factors such as female gender, lower education and younger age have been previously discussed as being connected to distress (González-Sanguino et al., 2020; Gibson et al., 2021; Santabárbara et al., 2021), another influential factor being household size (single person households) (Blix et al., 2021). Based on the empirical evidence of the pre-existing studies, it is hypothesized that psychological burden will increase concurrent to the severity of COVID-19 experience be it of self or other as well as closeness to the infected other, independent of the other risk factors.

## Method

### Sample Design

Data of the Survey of Health, Aging and Retirement in Europe (SHARE), a cross-national panel study, were used for analyses. Two Corona surveys were conducted by the SHARE, the second of which being released in February 2022. Data from the wave 9—COVID Survey 2—release version: 8.0.0 (Börsch-Supan, 2022), which was administered in 28 countries between June and August 2021 was used for the analyses in this study. Additionally, sociodemographic variables were imported from previous waves of the standard survey. Participants under 50 years and proxy interviews were excluded from the analyses resulting in a final sample of *n* = 46 129 persons which was made up of 59% female participants, mean age was 69.87 years (*SD* = 8.74 years, range 50–104 years) with 24% living alone during the pandemic. Education was distributed as follows: 32% low (ISCED 0–2), 44% middle (ISCED 3–4) and 24% high (ISCED 5–6) education. Comparing the study sample with the sample of the COVID survey 1 (summer of 2020) shows far larger prevalence rates of COVID-19 experiences in self and other, with rates rising multifold in all countries. A table providing comparative information between timepoints can be found in the Supplementary Table S1. Overall, 40.6% or respectively 18,757 of the 46,174 included participants indicated COVID-19 experience (self or other) in the COVID survey 2.

### Measures

To construct the dependent variable of psychological burden increase, data of multiple items (three cluster of questions) were combined. First, the emotional state of the participant was queried: feeling (1) nervous, anxious or on edge, (2) sad or depressed or whether they had (3) trouble sleeping in the last month (yes/no answer). Following, changes in frequency as compared to life during the first wave of the pandemic were asked (less so/about the same/more so). An additive index was constructed encompassing information how many emotional states were reportedly experienced with increased frequency (0–3) informing on overall psychological burden increase, a comprehensive concept (Staner, 2003; Nutt et al., 2008; Tiller, 2012) with moderate to strong intercorrelations between the measures (correlations ranged from Phi = 0.3 to 0.5 in the dataset). Finally, the index was dichotomized: (0) no or similar or lesser burden as compared to life during the first wave (summer 2020), (1) increased psychological burden.

The central predictive variable is COVID-19 experience which was defined as (a) the person's own experience with the virus or (b) an experience with the virus in the person's social network, categorized by severity: (0) no Covid-19 case, (1) positive Covid-19 test, (2) hospitalization or death^2^. In order to analyze the effects of COVID-19 experiences in the social environment on the respondents more thoroughly, a division into a “close”—spouse/partner, parent, child or other household member and a more “distant” network—other relative outside household, neighbor/friend/colleague, caregiver or other was performed. The resulting complex of variables was termed severity of COVID-19 experience (SoCE) as seen in Table 1. When multiple experiences were reported by the respondent, the most severe experience was used for analyses.

**Table 1:** Operationalization.

**Variable**	**Manifestation**	
Increased psychological burden	0 “no reported psychological burden or similar or lesser burden as compared to during the first wave” 1 “reported higher psychological burden in one or more adverse psychological states (nervous, anxious or on edge, and/or sad or depressed and/or trouble sleeping)”	
Severity of COVID-19 experience	Self	0 “No COVID-19” 1 “tested positive” 2 “hospitalized”
	close network/ distant network	0 “No COVID-19” 1 “Anyone tested positive for COVID-19” 2 “Anyone hospitalized/died due to COVID-19”
Health vulnerability	subjective health status prior to the pandemic	1 “excellent/very good” 2 “good” 3 “fair/poor”
	health status during the pandemic	0 “same or improved” 1 “deteriorated”
Variables describing subjective loss of control	postpone medical appointment refusal of medical appointment	0 “no” 1 “yes”
	receive help from children to obtain necessities since outbreak	
	receive help from others to obtain necessities since outbreak	
	able to make ends meet	0 “fairly/easily.” 1 “with some/great difficulty”
Sociodemographic variables	highest formal education	1 “low” (= ISCED 97; 0, 1 and 2) 2 “middle” (= ISCED 97; 3 and 4) 3 “high” (= ISCED 97; 5 and 6)
	household Size	0 “One-person household” 1 “Two-person household” 2 “3+ person household”
	Age	0 “50–64” 1 “65–79” 2 “80+”
	Gender	1 “male” 2 “female”

Control variables were included to adjust for additional burdens experienced during the pandemic. The following variables, which can be summed up into the three dimensions- health vulnerability, variables describing subjective loss of control and sociodemographic factors- were included into the model. Health vulnerability was depicted using two measures (a) subjective health status and (b) perceived change in health status compared to three months ago. Five variables were used to capture subjective loss of control during the pandemic. Two variables contained information on possible medical care shortages pertaining to (a) postponements of appointments (“Since your last interview/since July 2020, did you have a medical appointment scheduled, which the doctor or medical facility decided to postpone due to Corona?”)^3^ and (b) refusal of appointments (“…, did you ask for an appointment for a medical treatment and did not get one?”). Additionally, measures of social dependence were added with participants being asked whether (c) children or (d) other persons such as parents, relatives, friends or acquaintances were necessary to obtain goods or services during the pandemic (“Since the outbreak of corona, were you helped by the following people from outside your home to obtain necessities, e.g. food, medications or emergency household repairs?”). This division assumes that older persons report fewer issues when receiving help from their children as opposed to older (ex. parents) or more distant persons, according to the principle of reciprocity (Mancini and Simon, 1984). Finally, (e) participants were asked to inform on their financial situation during the pandemic (ability to make ends meet). Sociodemographic variables were also included. Level of formal education rated using the ISCED 1997 system and was divided into three categories (1) low (ISCED 0–2), (2) middle (ISCED (3–4) and (3) high (ISCED (5–6). Additionally, household size, (0) single-person household, (1) two-person household and (2) 3+ person household, age [(0) 50–64 years, (1) 65–79 years and (2) 80+ years] and gender [(1) male, (2) female] were included. For a detailed overview of the operationalization please refer to Table 1.

### Statistical Analysis

Data analysis was conducted using IBM SPSS 27. Unweighted data was used for analyses. Chi^2^ tests as well as Bonferroni adjusted *post-hoc* comparisons were computed to assess the correlations between burden and SoCE. To test the hypothesized influence of SoCE on psychological burden over and above all other variables, a logistic regression model was constructed using psychological burden as the dependent variable and introducing SoCE as well as all mentioned control variables as explanatory variables.

## Results

Table 2 provides information on increased burden across severity levels (positive test, hospitalization, test) while Table 3 looks at this association broken down for individual relationship groups affected by the virus (self, partner, children, etc.). Both tables include descriptive information on the phenomenon of interest, as well as the results of the bivariate analysis with burden. Most participants did not know anyone with a positive COVID-19 test result (64%), who was hospitalized (88.6%) or died due to COVID-19 (91.6%). While 14% of participants knew 3+ persons who had a positive test result, only a very small share of participants (0.8%) reported three or more deaths due to COVID-19 in their social circle. Correlative analyses showed a small (e.g. 19% to 22.8%, see Table 3) but significant association between respondent burden increase and the number of infected persons in the network. However, *post-hoc* analysis revealed the significant increase to be between no COVID-19 experience and at least one person with COVID-experience in the social network (for significant differences see subscript letters). Interestingly, the proportion of burdened respondents did not increase significantly with a rising number (e.g. 2 or more) of infected individuals in the network, suggesting a habituation effect.

**Table 2:** Descriptive and bivariate results of severity of COVID-19 experience and psychological burden increase.

	**Severity of Covid Experience**					
	**Tested positive**		**Hospitalization**		**Death**	
	**Prevalence %**	**Increased burden %**	**Prevalence %**	**Increased burden %**	**Prevalence %**	**Increased burden %**
No experience	64.0	19.0_a_	88.6	19.7_a_	91.6	19.7_a_
1 person	14.0	22.8_b_	7.9	26.7_b_	6.3	28.8_b_
2 persons	8.0	22.4_b_	2.2	23.4_b_	1.2	26.9_b_
3+ persons	14.0	23.2_b_	1.3	28.9_b_	0.8	27.8_b_
*n/p*	46,174	45,538/ <0.001	46,174	45,538/ <0.001	46,174	45,538/ <0.001

*Post-hoc tests are indicated with a,b,c with different letters informing on significant differences. n, number of cases, p, significance level, prevalence % refers to the extent of the described phenomenon (e.g. tested positive) in the sample*.

**Table 3:** Descriptive and bivariate results of severity of COVID-19 experience and increased psychological burden by relationship to affected party.

	**Relationship to the respondent**					
	**Self**		**Partner**		**Children**	
	**prevalence %**	**Increased burden %**	**prevalence %**	**Increased burden %**	**Prevalence %**	**Increased burden %**
No experience	93.3	19.8%_a_	95.0	20.1%_a_	88.4	20.2%_a_
positive test	5.4	27.4%_b_	4.1	24%_b_	11.0	22.1%_b_
hospitalization/death	1.1	40.7%_c_	0.9	44.7%_c_	0.5	34.2%_c_
*n/p*	46 123	45489/ <0.001	46114	45480/ <0.001	46114	45480/ <0.001
	**Other relatives**		**Neighbors and friends**		**Others**	
	**Prevalence %**	**Increased burden %**	**Prevalence %**	**Increased burden %**	**Prevalence %**	**Increased burden %**
No experience	80.8	19.8%_a_	84.4	19.9%_a_	98.1	20.4%_a_
positive test	13.6	21.8%_b_	8.4	22.9%_b_	0.9	23.9%_a_
hospitalization/death	5.7	26.6%_c_	7.2	23.8%_b_	1	24.1%_a_
*n/p*	46,114	45,480/ <0.001	46,114	45,480/ <0.001	46,114	45480/0.039

*Post-hoc tests are indicated with a,b,c with different letters informing on significant differences. n, number of cases, p, significance level, prevalence refers to the extent of the described phenomenon (e.g. tested positive) in the sample*.

Two effects can be reported for the impact of experience severity on burden increase when broken down to individual relationship groups: Proximity of the relationship affects the strength of correlation of infection severity and psychological burden. Significant correlations with increased burden were found for all levels of closeness but were smaller for more distant relations (e.g. compare partner 20.1–44.7% vs. neighbors/ friends 19.9–23.8%). COVID-19 experiences in persons closer to the participant seem to be more pivotal for increased burden. Additionally, larger prevalence for COVID-19 experiences can be seen for relationships potentially involving more persons (ex. more children than partners), this prevalence increase is found for close relationships (children, relatives) but is minimal in more distant relationships which may be due to less information on infection experience (and severity) being shared outside of the familial circle (Table 3). Summing up it can be reported that apart from own severe COVID-19 experience (hospitalization), psychological burden increase is affected by severe (hospitalization and death) but also moderate (positive test result) experiences of COVID-19 in the family.

The association between psychological burden increase and SoCE was further tested on its robustness in a multivariate model (logistic regression) introducing control variables. The sample included in the model was comprised of 43,025 observations due to missing values. With Nagelkerke's *R*^2^ at 0.147, the Hosmer-Lemeshow test at 0.204 and ROC AUC at 0.712; the model is deemed acceptable. Multicollinearity was examined and was also deemed acceptable. Odds ratios are presented in Table 4. SoCE—self, and distant network remained a significant predictor for psychological burden increase across all levels, with risk of increased burden being higher in more severe COVID-19 experience. For own experience, a positive test sufficed to have heightened risk of increased burden (*OR* = 1.253, CI 1.124–1.396), hospitalization led to an additional increase in probability of burden (*OR* = 1.684, CI 1.374–2.064)^4^. The overall highest effect on burden increase pertaining to SoCE was seen when a person close to the participant had a severe COVID-19 experience (hospitalized or died, *OR* = 1.940, CI 1.664–2.262) exceeding own infection experience. In the close network only hospitalization or death increased risk of burden significantly (*OR* = 1.405, CI 1.308–1.510). All variables of health vulnerability and subjective loss of control were significant predictors of psychological burden with deterioration of health being the strongest factor (*OR* = 2.990, CI 2.805–3.187). Being denied a medical appointment, which can be seen as a threat to subjective control over the situation, impacted psychological burden roughly as much as having a person from the more distant network be hospitalized or die due to the virus. As expected, being helped by others was a stronger predictor of burden than being helped by one's children. Male gender, having a moderate education level and being between 65 and 79 years old (as compared to 50 to 64 years old) were seen to be protective factors against psychological burden increase while household size did not significantly impact psychological burden over and above the other variables.

**Table 4:** Logistic regression model predicting increased psychological burden.

			***OR***	**95% CI**	**Wald statistic**	***p***
SoCE	COVID Self (ref. no COVID-19)					
	tested positive	**1.253**	1.124	1.396	16.515	<0.001
	hospitalized due to COVID-19	**1.684**	1.374	2.064	25.215	<0.001
	COVID Close Network (ref. no COVID-19)					
	Anyone tested positive	1.007	0.933	1.086	0.028	0.866
	Anyone hospitalized/died due to COVID-19	**1.940**	1.664	2.262	71.604	<0.001
	COVID distant Network (ref. no COVID-19)					
	Anyone tested positive	**1.193**	1.117	1.274	27.426	<0.001
	Anyone hospitalized/ died due to COVID-19	**1.405**	1.308	1.510	86.188	<0.001
Health vulnerability	Subjective health (ref. excellent/very good)					
	Good	**1.521**	1.401	1.652	100.049	<0.001
	fair/poor	**2.667**	2.453	2.899	529.418	<0.001
	Health change (ref. improve or same)	**2.990**	2.805	3.187	1130.017	<0.001
Subjective Loss of control	postpone medical appointment (ref. no)	**1.325**	1.235	1.421	61.427	<0.001
	denied medical appointment (ref. no)	**1.404**	1.260	1.564	37.922	<0.001
	receive help from children in obtaining necessities since outbreak (ref. no)	**1.100**	1.039	1.164	10.753	<0.001
	receive help from other in obtaining necessities since outbreak (ref. no)	**1.297**	1.207	1.394	49.878	<0.001
	able to make ends meet (ref. fairly/easily)	**1.322**	1.253	1.395	104.966	<0.001
SOCIO-demographic variables	Highest formal education (ref. low)					
	Middle	**0.934**	0.881	0.991	5.136	0.023
	High	**1.075**	1.002	1.152	4.050	0.044
	Household Size (ref. one-person)					
	two-person	1.018	0.957	1.084	0.336	0.562
	multi-person 3+	0.973	0.897	1.056	0.420	0.517
	Age (ref. 50–64)					
	65–79	**0.883**	0.832	0.938	16.197	<0.001
	80+	0.959	0.882	1.044	0.932	0.334
	Gender (ref. women)	**0.676**	0.641	0.713	205.726	<0.001
	*X^2^/df/p*	4239.830/21/ <0.001				
	Nagelkerkes R^2^	0.147				
	*N*	43,025				
	Hosmer-Lemeshow/ROC AUC	10.952; *p =* 0.204/0.712				

*Bold values signify statistically significant predictors of burden increase during the pandemic*.

## Discussion

The results of this study show increased psychological burden among persons 50+ during the pandemic in relation with COVID-19 experience in self or other. In summer of 2021 every fifth participant without COVID-19 experience (sample majority) reported increased burden, while this was true for every fourth who had experienced hospitalization and almost every third who had experienced a fatality due to the virus. Bivariate analysis revealed correlations between increased psychological burden and severity of experiences in all included relationship groups while the multivariate model informed on the effects robustness. The model revealed significant effects of both self- and other-experiences of COVID-19 illness over and above all other included potential stressors. Of the SoCE variables, severe experience of COVID-19 illness in the close social network was the strongest predictor of increased burden during the pandemic. This result is in line with previous findings, where fear for a close person's well-being has been reported to be a notable stressor (Bridgland et al., 2021; Richter and Heidinger, 2021a). It is interesting that COVID-19 experience “positive test” remained predictive of increased burden only for the distant circle and self. Own experience can be interpreted with the imminent threat to physical health. Potentially, well-being may be affected in cases in distant relations due to a lack of information on the persons resulting in a sense of powerlessness and heightened worry which may be less present in persons in the close network. An important caveat to the variable level “tested positive” is the missing information of the true severity of the infection, as it is likely that there were relatively severe cases of COVID-19 which were not treated in hospital. Therefore, it cannot be assumed that experiences classed in this section were mild and not traumatic, however most of these reported cases will have been milder than those requiring hospitalization or leading to death. Results shows the importance of multiple factors (health, social, financial, sociodemographic) which have significantly affected the psychological condition of the aged individual during the pandemic. Moreover, this study points to a culminative effect of these factors on psychological burden which demonstrate a clear and present need to gain control of the pandemic situation as adverse effects on well-being in the present may have larger, long-term effects on mental health in the elderly.

Limitations of this study are its cross-sectional design which restricts assumptions of causality.

The construction of the variable psychological burden increase can be criticized, as it is based on several standalone items measuring adverse states and changes based on subjective perception of the respondents. Unfortunately, no appropriate pre-pandemic measure of burden was available to control for baseline burden. This may be criticized, as persons with poor mental health prior to the outbreak of the pandemic were more likely to have increased mental issues during this time (Neelam et al., 2020). Furthermore, the available change measures were retrospective comparative assessments which may have introduced a recall bias. In addition, social network types are broadly defined to compensate for wildly fluctuating numbers. Fortunately, it must be said, there were few close network deaths, for example, in the data of summer 2021. Social network size may have influenced psychological burden as a larger network could have increased the possibility of knowing someone with COVID-19 experience and therefore may have led to increased psychological burden. Unfortunately, this variable was not available in the key surveys and was therefore not included into the model. This study uses pooled data of multiple European countries aiming to assess the impact of illness experience across Europe rather than within individual countries. This may have impacted the outcome as countries did have heterogeneous COVID-19 strategies. However, this study provides first evidence on the psychological impact of social network experience and may serve as a jumping off point to further in-depth analyses for individual countries. Despite these limitations, the study clearly shows that the pandemic has been detrimental to mental health with COVID-19 experience (infections, hospitalizations, and deaths) contributing to, but varying in intensity depending on social network proximity, psychological burden. This study expands upon previous work conducted in this field and reiterates the importance of regaining control of the pandemic.

## Data Availability Statement

Publicly available datasets were analyzed in this study. This data can be found online at: https://doi.org/10.6103/SHARE.W9CA.800.

## Ethics Statement

Ethical review and approval was not required for the study on human participants in accordance with the local legislation and institutional requirements. The patients/participants provided their written informed consent to participate in this study.

## Author Contributions

TH was the lead author on this paper. LR provided the analysis. Both authors contributed to the article and approved the submitted version.

## Funding

This work was supported by Karl Landsteiner University of Health Sciences in Krems, Austria.

## Conflict of Interest

The authors declare that the research was conducted in the absence of any commercial or financial relationships that could be construed as a potential conflict of interest.

## Publisher's Note

All claims expressed in this article are solely those of the authors and do not necessarily represent those of their affiliated organizations, or those of the publisher, the editors and the reviewers. Any product that may be evaluated in this article, or claim that may be made by its manufacturer, is not guaranteed or endorsed by the publisher.

## Footnotes

^1^Experienced psychological burden may have been due to own rather than other infection (transmission).

^2^Logically occurrence of death due to the virus was only surveyed for the other-experience.

^3^Persons who were not interviewed in Corona survey 1 were asked to compare to July 2020.

^4^Due to a minimal overlap of the CIs of these levels, additional testing with changed reference category was undertaken which showed a significant difference between self-positive COVID-19 test- and self-hospitalization on burden increase (*p* = 0.009). This can be seen as clear indication, that these circumstances do impact burden increase differently.
