# Supplementary material for: Examining the Impact of COVID-19 Experiences on Reported Psychological Burden Increase in Older Persons: The Effects of Illness Severity and Social Proximity
Source: Front Psychol. 2022 May 13;13:884729. doi: 10.3389/fpsyg.2022.884729 (PMC9136394; doi:10.3389/fpsyg.2022.884729)
Supplement: Supplementary file 1 [file Data_Sheet_1.PDF]

Table S1- Covid-19 experiences across European countries subdivided between self and other as well as experience severity

	SHARE Covid Survey 1						SHARE Covid Survey 2					
	self		network			*n*	self		network			*n*
	positive	hospitalized	positive	hospitalized	passed away		positive	hospitalized	positive	hospitalized	passed away	
Luxembourg	0,9%	0,0%	13,60%	6,00%	4,80%	870	7,9%	0,7%	35,10%	11,20%	13,50%	*806*
Belgium	0,6%	0,4%	8,70%	5,20%	10,20%	3730	5,8%	1,5%	26,50%	7,80%	14,10%	*3318*
Sweden	0,9%	0,4%	11,60%	4,80%	4,80%	1377	4,0%	0,7%	35,9%	7,0%	8,5%	*957*
Netherlands	0,3%	0,1%	7,20%	4,40%	8,40%	797	5,3%	0,6%	32,80%	8,40%	9,00%	*722*
Spain	0,6%	0,3%	6,20%	4,90%	7,40%	1811	5,3%	1,4%	20,60%	7,90%	10,70%	*1511*
Switzerland	0,50%	0,20%	8,40%	5,00%	4,50%	1893	4,1%	1,1%	26,00%	10,80%	11,20%	*1696*
Denmark	0,8%	0,1%	9,90%	2,60%	1,70%	2001	2,4%	0,4%	31,30%	3,40%	1,90%	*1573*
Austria	0,6%	0,2%	8,4%	3,2%	2,9%	2462	3,8%	0,8%	25,30%	9,10%	12,30%	*2126*
Portugal	0,3%	0,2%	10,30%	2,00%	2,30%	989	3,7%	0,5%	27,90%	7,00%	10,60%	*924*
France	0,7%	0,4%	6,70%	3,20%	3,80%	2028	3,0%	0,8%	22,80%	6,10%	7,50%	*1783*
Italy	0,60%	0,10%	2,80%	3,20%	7,60%	3557	4,9%	0,9%	14,80%	5,20%	12,00%	*3171*
Israel	0,2%	0,1%	5,10%	2,20%	3,00%	1345	3,0%	0,4%	19,40%	5,20%	9,90%	*1116*
Germany	0,60%	0,10%	5,10%	1,70%	1,60%	2699	2,20%	0,70%	19,90%	5,30%	6,10%	*1968*
Malta	0,4%	0,0%	3,90%	1,80%	1,90%	795	5,00%	1,20%	22,00%	3,20%	4,90%	*677*

Note. The included countries reported prevalence rates of Covid-19 experiences of 5% at minimum in summer of 2020.
